# The Impact of an Incentive on the Use of an Online Self-Directed Wellness and Self-Management Program

**DOI:** 10.2196/jmir.3239

**Published:** 2014-10-02

**Authors:** Judith H Hibbard, Jessica Greene

**Affiliations:** ^1^University of Oregon-1209Health Policy Research GroupEugene, ORUnited States; ^2^George Washington UniversitySchool of NursingWashington, DCUnited States

**Keywords:** empowerment, health promotion, Internet, incentives, patient activation

## Abstract

**Background:**

Those who pay for health care are increasingly looking for strategies to influence individuals to take a more active role in managing their health. Incenting health plan members and/or employees to participate in wellness programs is a widely used approach.

**Objective:**

In this study, we examine financial incentives to health plan members to participate in an online self-management/wellness program—US $20 for completing the patient activation measure (PAM) and an additional US $40 for completing 8 learning modules. We examined whether the characteristics of plan members differed by the degree to which they responded to the incentives. Further, we examined whether participation in the wellness program was associated with improvements in PAM scores and changes in health care utilization.

**Methods:**

This retrospective study compared demographic characteristics and change in PAM scores and health utilization for 144,625 health plan members in 2011. Four groups were compared: (1) those who were offered the incentives but chose not to participate (n=128,634), (2) those who received the initial incentive (PAM only) but did not complete 8 topics (n=7099), (3) those who received both incentives (completing 8 topics but no more) (n=2693), and (4) those who received both incentives and continued using the online program beyond what was required by the incentives (n=6249).

**Results:**

The vast majority of health plan members did not participate in the program (88.91%, 128,634/144,675). Of those who participated, only 7099 of 16,041 (44.25%) completed the PAM for the first incentive, 2693 (16.79%) completed 8 topics for the second incentive, and 6249 (38.96%) received both incentives and continued using the program beyond the incentive requirements. Nonparticipants were more likely to be men and to have lower health risk scores on average than the other three groups of participants (*P*<.001). In multivariate regression models, those who used the online program (8 topics or beyond) increased their PAM score by approximately 1 point more than those who only took the PAM and did not use the wellness program (*P*<.03). In addition, emergency department visits were lower for all groups who responded to any level of the incentive as compared to those who did not (*P*<.01). No differences were found in other types of utilization.

**Conclusions:**

The incentive was not sufficient to spark most health plan members to use the wellness program. However, the fact that many program participants went beyond the incentive in their use of the online wellness program suggests that the users of the online program found value in using it, and it was their own internal motivation that stimulated this additional use. Providing an incentive for program participation may be an effective pathway for working with less activated patients, particularly if the program is tailored to the needs of the less activated.

## Introduction

### Theory and Prior Work

Patients are key in determining their health care outcomes. They are the ones that carry out the day-to-day care management tasks and decide whether or not to make the necessary lifestyle adjustments to improve their health. Without patients’ participation, even with best practices on the part of health care providers, it is very difficult to achieve optimal health outcomes [1]. To control costs and improve outcomes, insurance companies, government, and employers are increasingly looking for strategies to influence individuals to take a more active role in managing their health.

Incenting health plan members and/or employees to take Health Risk Appraisals (HRAs) is a widely used approach. The HRAs typically include questions about health behaviors and provide the individual feedback with a list of behavioral changes they could make to improve their health. A 2013 study estimated that 55% of large employers offered an HRA to employees and 54% of those provided an incentive to complete it [2]. That was up from 49% offering the HRA five years ago in 2008 and 33% incenting it. The use of HRAs is based on the assumption that raising awareness of health risks and needed behavioral changes will be sufficient to stimulate change, an assumption that is often not borne out [3]. Now the trend has expanded to include incentives that are tied to participation in a wellness program. For example, the 2013 Health Research and Educational Trust (HRET) survey found that 99% of large firms offer wellness programs and 36% incent participation in them [2]. Towers Watson Staying@Work Survey Report found that in 2011, half of employees surveyed were offered financial rewards for participation in a health program [4]. Under the Affordable Care Act (ACA) in the United States, new rules will allow employers to increase the use of incentives for wellness programs [5].

Studies indicate that incentives are most effective at achieving behavior change that requires a single activity, such as receiving a vaccination or a screening, as compared to actions that require ongoing engagement [6,7]. There is concern that when health plans or insurers offer incentives, discrimination or disadvantages do not accrue to those who choose not to participate. Of particular concern is when a specific health behavior is the focus, for example smoking, that the individual who opts out of participation is not penalized at work or in their access to insurance [8].

However, there are fewer concerns about the use of incentives to complete an HRA. There is evidence that incentives do yield greater participation in HRAs [9,10]. The degree to which incentives stimulate meaningful engagement in longer-term health programs is less clear. There is concern that financial incentives may supplant or undermine internal motivations. The worry is that the incentive may undermine a sense of personal responsibility for health and an individual’s intrinsic motivations to promote their own health [11].

Might an incentive to participate in a wellness/self-management program undercut internal motivation? One way to examine this question is to assess the impact of an incentive on program participation and on changes in patient activation. Patient activation is defined as having the knowledge, skill, and confidence to manage one’s own health and health care. Individuals who are more activated, are typically more motivated to effectively manage their health. While there are alternative ways to define activation or engagement, there is only one validated measure, the 13-item Patient Activation Measure (PAM). Multiple studies show that people who score higher on PAM are more motivated to improve their health. The PAM score is predictive of most health behaviors, many clinical indicators, and the use of costly health care services [12,13]. If PAM scores were to improve as a result of incented program participation, this would indicate that the incentive did not undercut internal motivation.

Most of the research on the efficacy of health program participation is based on programs offered at the worksite or programs offered in the community. Because wellness and self-management support programs are now also being offered as online programs, they are no longer limited to these venues. Studies indicate that there is some positive impact of self-directed online self-management programs. For example, Solomon and colleagues, evaluating the impact of an online program among chronic disease patients, found that the program increased patient activation an average of 4 points (on a 100-point scale) more than did the control group [14]. Similarly, Lorig and colleagues found that an online self-management program improved clinical outcomes and patient activation [15]. However, there appears to be no evidence on the value of offering incentives to participate in these self-directed online programs.

### Research Questions

In this study, we examine the impact of providing financial incentives to commercially insured health plan members to participate in an online self-directed self-management/wellness program. We assess the impact of a US $20 financial incentive to take a PAM assessment and a second US $40 incentive to complete 8 user-selected learning modules or topics in the online program and to set 8 behavioral goals.

Our specific research questions were: (1) What are the characteristics of those who respond to the two levels of financial incentives and those who do not respond at all to the incentive?; (2) What are the characteristics of those who participate beyond the financial incentive in using the wellness/ self-management program? How much further beyond the incentive does their participation go?; and (3) What is the impact of participating in the online program on patient activation and health care utilization?

## Methods

### Incentive

In 2011, commercially insured members of a large health plan were offered a US $20 incentive to go online to take the PAM questions. They were given a further US $40 incentive to complete 8 topics and set behavioral goals in the online self-directed wellness/self-management program. The participant could choose the topic areas and behavioral goals they wanted to focus on. Thus there are four groups in the study: (1) nonparticipants in the online program, (2) those who only completed the PAM and received the first incentive, (3) those who completed the PAM and 8 topics and received the first and second incentive, and (4) those who received both incentives and continued using the online program beyond the required number of topics.

### Online Wellness Program

Participants were incented to go to an online program that assessed their level of patient activation and to participate in a self-directed online wellness/self-management program. All the topics and suggested goals that users are able to select from in the online program are tailored to the users’ level of activation. The program tailors to the 4 levels of activation, from low (level 1) to high (level 4). Users can choose what they want to focus on, from disease specific self-management to wellness activities. The lowest activation level topics start at a very foundational level. The program breaks information down into small bits and suggests action steps or goals that less activated individuals are likely to succeed at, but may not be clinically meaningful at this point (eg, cut out fast food lunches 2 days a week). The approach is based on the theories of behavioral activation that show motivation will follow action [16]. The strategy is to start the individual acting, experiencing small successes, and those successes lead to greater motivation and confidence. Level 4 topics assume both a higher level of knowledge and skill and encourage users to set behavioral goals that, for example, help them stay on track with behaviors they have already adopted. Users focus on the topics they are interested in and set behavioral goals related to the topics they pursue. The program provides insights and strategies for overcoming barriers to meeting their goals.

### Design and Analysis

This retrospective study compared demographic characteristics and change in PAM and health utilization for four groups of health plan members in 2011: (1) those who were offered the incentives but chose not to participate in the online wellness program, (2) those who received the initial incentive (PAM only) but did not complete 8 topics and goals, (3) those who received both incentives (completing 8 topics and 8 goals but no more), and (4) those who received both incentives and continued using the online wellness program beyond what was required by the incentives. The study protocol (designated as 12012011.001), was approved by the internal review boards (IRB) of the University of Oregon and George Washington University.

### Study Population

**Figure 1:**
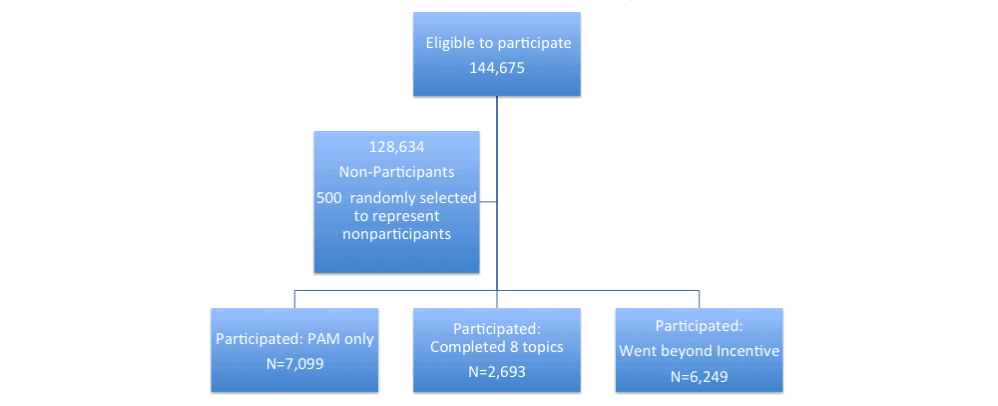
Study design showing the study groups.

The total population eligible for participation was 144,675 health plan members who were adults and had consistent coverage over the study period. Given that 128,634 people declined to respond to the incentive (88.91% of the eligible population did not use the online wellness program), we selected a random sample of 500 of these nonparticipating plan members to represent the nonparticipant group. The other 3 groups included all of those who chose to respond to the incentives and individually decided what level of incentive they would pursue (Figure 1). Observations are weighted in all analyses using the inverse probability of selection in order to reflect the percent of the total population they represent.

The study population excluded those plan members who were recruited for a special program that involved telephonic coaching because of serious health issues. We excluded this group whether they participated in the telephonic coaching or not. These excluded patients were sicker with more chronic conditions and higher levels of hospitalization and emergency department use than the larger population of plan members.

### Measures

The independent variable in this study was the extent to which the eligible health plan member used the online wellness program (no participation, PAM only/low incentive, completed 8 topics/both incentives, went beyond incentive requirements).

Dependent variables included the PAM and health care utilization measures. The PAM consists of 13 items that form an interval level, unidimensional, Guttman-like scale. The measure has been shown to have strong psychometric properties. The PAM items are statements on confidence, beliefs, knowledge, and skills about managing one’s health, which respondents can answer with degrees of agreement or disagreement (eg, I know how to prevent problems with my health; I am confident that I can tell a doctor my concerns, even when he or she does not ask). The measure is scored on a theoretical 0-100 scale with most patients falling in the 35-95 point range. Four levels of activation have been previously identified, reflecting a developmental progression from passive receipt of care toward greater activation [17,18].

Utilization data were derived from claims data. Primary care, specialty care, emergency department (ED) use, and hospital stays were the utilization variables assessed. For primary and specialty care visits, we examined the actual number of visits in the pre and post period. For the ED and hospitalizations, we examined whether or not there were any visits.

In our analyses we also examined and controlled for demographic and health variables. Average income for the member’s zip code is used as a proxy for member’s income in the analysis. The Adjusted Clinical Groups (ACG) score is a measure of illness severity and is included in the analysis to control for health status differences in the study groups.

### Analytic Approach

We first examined the demographic and health characteristics of the four groups, using baseline data from the last 6 months of 2010, using chi-square and analysis of variance (ANOVA) tests. Additionally, we used descriptive statistics to examine how much use of the online wellness program each of the four groups engaged in.

Then, we examined bivariate relationship between the changes from pre- to post- in the outcome measures (PAM scores and utilization) using follow-up data collected from the first 6 months of 2012. Finally, we developed multivariate analyses to examine the relationships between group participation level and the post-period outcomes, controlling for the pre-period outcome value, demographic characteristics, and ACG risk score. The sample size for Patient activation in the follow-up is reduced to 46% of the original sample size because not all members completed the pam again in 2012. All analyses weight the observations by the inverse probability of selection and adjust the standard errors accordingly.

## Results

### Characteristics of Those Who Responded to Each Level of the Incentive

Table 1 shows that the vast majority of study participants did not use the online wellness program at all (88.91%, 128,634/144,675). Of the 16,041 people who did use it, 7099 (44.25%) people participated only enough to receive the initial incentive, 2693 (16.79%) received the second higher incentive, and 6249 (38.96%) went beyond the incentive requirements.

The people who participated in the four different levels of the online wellness program had different demographic characteristics (Table 1). The group that did not participate in the online wellness program had a higher percent of males than the groups that did respond, and women were more likely to comprehensively use the online program. There were also age differences in who and how much members responded to the incentive. Those that responded to the higher incentive only were slightly younger than those in the other groups. The average income (based on zip code) was not different for the four groups. People whose online program use went beyond the incentive requirements had a slightly higher baseline PAM score than did those who did only what the incentives required (there were no PAM scores on the group that did not participate in the online program at all).

There were also differences in the groups in terms of their health status. Those who responded to the incentive or went beyond the incentive had slightly higher ACG risk scores than those not responding to the incentive. However, those not responding to any of the incentives were more likely to have diabetes and hypertension than those who responded to the incentive or went beyond the incentive.

It is interesting to note that those members who went beyond the incentive in their use of the online program, had usage considerably beyond the requirements of the incentive, viewing on average over 20 topics (Table 2); 69.88% (6249/8942) of those who got the maximum incentive, went beyond what the incentive required.

We also looked at baseline PAM level as a predictor of how members responded to the incentive. Table 3 shows percent of members at each level of activation at baseline and their distribution over the three groups responding to the incentive (no PAM scores were available for those who did not respond to the incentive so they are not included in this analysis). Members at the lowest level of activation at baseline were slightly more likely to take the minimum incentive (PAM only), than those at the higher levels of activation. However, even among the least activated, 32.0% (231/723) went beyond the incentive in their use of the online program, while 40.50% (2828/6983) of the highest activated went beyond the incentive.

**Table 1:** Demographic and health characteristics of the study sample, by program participation level.

Demographic/characteristic		Total sample^a^ (n=16,493)	Subsample of non-participants^a^ (n=452)	PAM^b^ only: Low incentive participation (n=7099)	Completed 8 topics: High incentive (n=2693)	Went beyond incentive requirements (n=6249)	*P* value
Weighted percent of population		-	14,609 (88.58%)	870 (5.27%)	305 (1.85%)	709 (4.30%)	
**Gender, n (%)**							
	Male	8881 (53.85)	8048 (55.09)	366 (42.06)	153 (50.16)	314 (44.29)^c^	<.001
	Female	7612 (46.15)	6561 (44.91)	504 (57.93)	152 (49.84)	395 (55.71)^c^	
Age, mean (SD)		41.3 (13.4)	41.2 (13.5)	42.8 (12.2)	39.2 (11.8)	41.9 (11.6)^c^	<.001
Income of zip code, mean (SD)		23,967.34 (7131.31)	23,947.40 (7164.20)	24,318.16 (6899.29)	24,137.45 (7199.90)	23,874.46 (6846.15)	.48
PAM score, mean (SD)		N/A	N/A	70.3 (15.3)	70.5 (15.0)	71.7 (15.3)^c^	<.001
Health Risk Score, mean (SD)		2.0 (1.3)	1.9 (1.3)	2.1 (1.2)	2.1 (1.2)	2.2 (1.1)^c^	<.001
**Chronic conditions, n (%)**							
	Depression	2156 (13.07)	1939 (13.27)	103 (11.84)	30 (9.84)	83 (11.71)	.24
	Diabetes	519 (3.15)	485 (3.32)	15 (1.72)	5 (1.64)	14 (1.97)^a^	.02
	Hypertension	2307 (13.99)	2101 (14.38)	93 (10.69)	28 (9.18)	85 (11.99)^a^	.02

^a^Since we examined a random sample of people who never participated in online coaching, the descriptive statistics are weighted using the inverse probability of selection for the no coaching group, and the standard errors are weighted accordingly. The numbers presented in the table for percentages reflect the weighted numbers.

^b^PAM: patient activation measure

^c^Chi-square statistical tests were used for categorical demographic and health variables (gender and having specific chronic conditions), while ANOVAs were used for continuous variables (age, mean income of zip code, health risk score).

**Table 2:** Incentive response and use of online wellness/ self-management program.

Program use	PAM^a^ only: Low incentive	Completed 8 topics: High incentive	Went beyond incentive requirements	ANOVA
	mean (SD)	mean (SD)	mean (SD)	*P* value
Number of health topics accessed	1.2 (2.2)	8.6 (1.0)	20.2 (18.4)	<.001
Number of health topics completed	0.9 (1.9)	8.0 (0.0)	19.4 (18.2)	<.001
Number of months of use	1.2 (0.6)	1.4 (0.8)	1.7 (1.1)	<.001

^a^PAM: patient activation measure

**Table 3:** Patient activation level and response to incentive.^a^

Response	PAM^b^ Level 1 (n=723)	PAM Level 2 (n=991)	PAM Level 3 (n=7342)	PAM Level 4 (n=6983)
	n (%)	n (%)	n (%)	n (%)
PAM only: Low Incentive	388 (53.67)	445 (44.90)	3237 (44.09)	3027 (43.35)
Completed 8 topics: High Incentive	106 (14.66)	179 (18.06)	1282 (17.46)	1126 (16.12)
Went beyond incentive requirements	229 (31.67)	367 (37.03)	2823 (38.45)	2830 (40.53)

^a^Chi-square *P* value <.001

^b^PAM: patient activation measure

### Impact of Intervention on Patient Activation and Utilization

Table 4 shows the bivariate relationships between the program participation level and the pre, post, and change in outcome variables. The results indicate that there is a significant difference in PAM scores in the pre and post periods across the three groups who used the online program (we did not have PAM data on those who did not participate); however, the difference in the increase from baseline to follow-up is not significant.

There were no significant differences in the groups in primary care, specialty care, or hospital visits in either the baseline or post period. However, there were differences in the groups in the post period for ED visits, with highest rates for nonparticipants. It is notable that ED visits increased for all groups from the baseline to the post period, but the change was not significantly different among the groups.

Table 5 shows the multivariate regression results, which control for baseline outcome value, age, gender, income, and baseline ACG risk score. In this analysis, patient activation scores at follow-up are significantly greater (by approximately 1 point) for those who used the online wellness program as compared to those who only took the PAM. In addition, use of the ED is significantly lower for all groups who responded to any level of the incentive as compared to those who did not participate. No differences were found in other types of utilization.

Table 6 shows how much patient activation scores changed among those who made any use of the online program. The changes in scores are shown within baseline PAM levels. At baseline, those who scored in the lower two levels of patient activation increased their PAM score the most following participating in the online program. Members who at baseline scored in the lowest level of activation gained an average of 21 points (on a 0-100 scale).

**Table 4:** Bivariate relationships between utilization, cost, and participation level.

		No participation	PAM^c^ only: Low incentive participation	Completed 8 topics: High incentive	Went beyond incentive requirements	*P* value
**Patient activation score** ^a^ **, mean (SD)**						
	Pre-period	n/a	70.7 (14.6)	71.2 (14.7)	72.1 (15.3)	.002
	Post-period	n/a	72.4 (14.6)	73.6 (15.1)	74.1 (16.2)	<.001
	Change	n/a	1.7 (4.8)	2.5 (15.5)	2.0 (16.3)	.33
**Primary care visits, mean (SD)**						
	Pre-period	2.4 (5.4)	2.4 (4.8)	2.4 (4.6)	2.6 (5.0)	.47
	Post-period	3.1 (4.9)	3.5 (5.3)	3.1 (5.6)	3.4 (5.9)	.06
	Change	0.7 (6.8)	1.0 (6.5)	0.7 (6.9)	0.8 (7.1)	.50
**Specialty care visits, mean (SD)**						
	Pre-period	2.9 (7.2)	2.8 (6.5)	2.8 (5.9)	2.9 (6.4)	.86
	Post-period	3.7 (9.1)	4.1 (8.0)	3.6 (7.3)	3.9 (7.9)	.10
	Change	0.8 (9.5)	1.3 (8.7)	0.8 (8.3)	1.0 (8.9)	.20
**Emergency department visits, n (%) with a visit** ^b^						
	Pre-period	635 (4.5)	27 (3.0)	9 (3.1)	24 (3.4)	.41
	Post-period	1103 (7.8)	33 (3.7)	12 (4.0)	31 (4.3)	.01
	Change	3.3 (0.3)	0.7 (0.3)	0.9 (0.3)	0.9 (0.3)	.40
**Hospitalizations, n (%) with a visit** ^b^						
	Pre-period	226 (1.6)	10 (1.1)	3 (1.0)	8 (1.2)	.81
	Post-period	291 (2.0)	17 (2.0)	7 (2.2)	16 (2.2)	.83
	Change	0.4 (0.2)	0.8 (0.2)	1.2 (0.2)	1.1 (0.2)	.74

^a^The sample size for PAM was n=2222 for PAM only, n=1522 completed 8 topics, and n=3894 for going beyond the incentive requirements.

^b^The numbers presented in the table for percentages reflect the weighted numbers.

^c^PAM: patient activation measure

**Table 5:** Key regression coefficients from models examining follow-up PAM^a^ and health care utilization, by participation status.^b^

Participation	PAM		Primary care visits		Specialty care visits		Any ED^c^ visits		Any hospitalizations	
	Coeff	*P* value	Coeff	*P* value	Coeff	*P* value	Coeff	*P* value	Coeff	*P* value
No Participation	n/a		--		--		--		--	
PAM only: Low incentive participation	--		.01	.97	.16	.73	−.05	.005	.00	.62
Completed 8 topics: High incentive only	.94	.03	−.14	.59	−.21	.65	−.05	.007	.01	.26
Went beyond incentive requirements	.99	.005	−.05	.86	−.04	.93	−.04	.01	−.01	.28

^a^PAM: patient activation measure

^b^Models control for baseline value of the dependent variable, gender, age, terciles of mean income of zip code, and baseline Adjusted Clinical Groups (ACG) risk score.

^c^ED: emergency department

**Table 6:** PAM^a^ score changes among those responding to incentive.

	PAM Level 1 at baseline	PAM Level 2 at baseline	PAM Level 3 at baseline	PAM Level 4 at baseline	*P* value
	mean (SD)	mean (SD)	mean (SD)	mean (SD)	
Any use of online program	21.0 (22.2)	11.5 (13.1)	5.2 (13.0)	−4.0 (15.1)	<.001

^a^PAM: patient activation measure

## Discussion

### Principal Findings

Only about 11% of those offered an incentive to participate in an online wellness program participated in it. Those who participated in any level of the incentive were more likely to be female, slightly older, and less likely to have diabetes, hypertension, and depression than those who did not respond. We did not have PAM data on the group who did not respond to the incentive; however, among those who did respond, those with slightly higher scores at baseline were more likely to go beyond the incentive in using the online program. That is to say, the incentive seemed to be most effective in stimulating participation among those who were slightly more activated and in better health. This is consistent with a prior study indicating that it is the more activated who are more likely to participate in offered health programs [19]. It is also consistent with findings that suggest that incentives are more effective when the incentive does not require difficult or sustained behavior change [7].

Contrary to concerns raised by some investigators, the findings do not indicate that the incentive to participate in the online program undermined or supplanted internal motivations to manage one’s own health [11]. For those who used the online wellness program, 40% went beyond what the incentive required, and 70% of those who took the maximum incentive, continued to use the program beyond the incentive. This suggests that the users of the online program found value in using it, and it was their own internal motivation that stimulated this additional use. Further, it appears that participation in the online program increased PAM scores, particularly among those who began at the lower two levels of activation. The findings also show a lesser increase in emergency department use for those who participated in any way in the program compared to those who did not use the online wellness program. Given that the sickest and costliest patients were excluded from the analysis (those invited to telephonic coaching were excluded), it is somewhat surprising that this type of “self-directed” intervention would impact utilization among those who are moderate to low health care utilizers.

The findings break new ground in this research arena, in that most evaluations of wellness programs and incentives do not measure the impact of the program on the individual’s knowledge, skill, and confidence in managing their health (activation). By including activation in the analysis, the findings help to elucidate who these programs are reaching (eg, are they reaching the more or less activated?) and who they are helping.

### Limitations

At the same time, the findings are limited by the fact that we did not have PAM data on those who chose not to participate in the incentive program, nor on participants who did not complete a PAM in 2012 as well as 2011. They are also limited by the design of the study. Because participants were not randomly assigned to the four study groups, we cannot rule out that the findings are the result of some other, unmeasured, factor. Finally, because the study was conducted within one health plan in one geographic region and only included insured adults, the generalizability of the findings are limited.

The findings indicate that participantion in the program resulted in a small decline in the PAM scores of those who, at baseline, were in the highest level of activation. It may be that the intervention was not as useful or appropriate for these participants. However, the fact that program participation appeared to stimulate increased activation in those who were the least activated at baseline is a promising finding. These findings are consistent with the findings from other intervention studies that show that the least activated gain the most from an intervention [14,15,20]. Research shows that increases in activation are related to improvements in health behaviors, clinical indicators, and reductions in costly health care utilization [1]. As we move into an era where organizations are held accountable for costs and outcomes, finding ways to engage the least activated is becoming a priority.

### Conclusions

While the incentive was most effective in recruiting higher activated members, less activated members also responded to the incentive. Providing an incentive for program participation may be an effective pathway for working with less activated patients, particularly if the program is tailored to the needs of the less activated. However, it is important to note that the vast majority of health plan members did not respond to the incentive at all.

With increased costs and an aging population base, strategies to effectively manage population health is a global concern. This study adds to our understanding of who is likely to respond to incentives and how participation in the programs may influence behaviors and health care utilization.

## Abbreviations

ACG: Adjusted Clinical Groups score
ED: emergency department
HRA: health risk appraisal
PAM: patient activation measure
